# Kinetics and Interrelations of the Renin Aldosterone Response to Acute Psychosocial Stress: A Neglected Stress System

**DOI:** 10.1210/clinem/dgz190

**Published:** 2019-11-10

**Authors:** Angelina Gideon, Christine Sauter, Judy Fieres, Thilo Berger, Britta Renner, Petra H Wirtz

**Affiliations:** 1 Biological Work and Health Psychology, University of Konstanz, Germany; 2 Health Psychology, University of Konstanz, Germany; 3 Centre for the Advanced Study of Collective Behavior, University of Konstanz, Germany

**Keywords:** acute psychosocial stress, renin, aldosterone, saliva, plasma, cortisol

## Abstract

**Context:**

The renin-angiotensin-aldosterone system (RAAS) plays an important role in cardiovascular homeostasis and its dysfunction relates to negative health consequences. Acute psychosocial stress seems to activate the RAAS in humans, but stress kinetics and interrelations of RAAS parameters compared with a nonstress control group remain inconclusive.

**Objective:**

We systematically investigated in a randomized placebo-controlled design stress kinetics and interrelations of the reactivity of RAAS parameters measured in plasma and saliva to standardized acute psychosocial stress induction.

**Methods:**

58 healthy young men were assigned to either a stress or a placebo control group. The stress group underwent the Trier Social Stress Test (TSST), while the control group underwent the placebo TSST. We repeatedly assessed plasma renin, and plasma and salivary aldosterone before and up to 3 hours after stress/placebo. We simultaneously assessed salivary cortisol to validate successful stress induction and to test for interrelations.

**Results:**

Acute psychosocial stress induced significant increases in all endocrine measures compared with placebo-stress (all *P* ≤ .041). Highest renin levels were observed 1 minute after stress, and highest aldosterone and cortisol levels 10 and 20 minutes after stress, with salivary aldosterone starting earlier at 1 minute after stress. Renin completed recovery at 10 minutes, cortisol at 60 minutes, salivary aldosterone at 90 minutes, and plasma aldosterone at 180 minutes after stress. Stress increase scores of all endocrine measures related to each other, as did renin and cortisol areas under the curve with respect to increase (AUCi) and salivary and plasma aldosterone AUCi (all *P* ≤ .047).

**Conclusions:**

Our findings suggest that in humans acute psychosocial stress induces a differential and interrelated RAAS parameter activation pattern. Potential implications for stress-related cardiovascular risk remain to be elucidated.

The renin-angiotensin-aldosterone system (RAAS) plays an important role in cardiovascular homeostasis by regulating maintenance of arterial blood pressure, water balance, and electrolyte homeostasis (1,2). Dysfunction of the RAAS relates to a variety of adverse health consequences including cardiovascular disease (CVD) and risk factors (2,3). Activation of the RAAS starts with renin release and ends with aldosterone secretion. Renin is an enzyme that is synthesized by juxtaglomerular cells in the kidney and released into the bloodstream. There, renin catalyzes hydrolysis of angiotensinogen, which is produced in the liver and in fat tissues, to angiotensin I (ANG-I). Via the blood circulation, ANG-I reaches the lung capillaries, endothelial cells, and kidney epithelial cells where angiotensin-converting enzyme (ACE) transforms ANG-I to ANG-II (2). ANG-II in turn induces aldosterone secretion in the zona glomerulosa of the adrenal cortex (1). Aldosterone is an adrenal corticosteroid hormone that is primary responsible for the regulation of sodium-potassium homeostasis by increasing sodium reabsorption in the kidney tubules, thus retaining Na^+^ and reducing K^+^ (4). Elevated aldosterone levels have not only been linked to renal fibrosis, but also to cardiovascular-related outcomes including cardiac fibrosis, inflammation, vascular remodeling, endothelial dysfunction (4,5), vasoconstriction, or insulin resistance (5).

Increasing evidence suggests that psychosocial stress is an independent risk factor for CVD (6,7). In particular, stress-induced hyperactivation of stress-responsive physiological systems has been proposed to play a role in the development of CVD. While the responsivity of stress hormones and intermediate biological risk factors such as coagulation, inflammation, or blood lipids to acute psychosocial stress induction is well documented (8–11), comparably little is known regarding stress reactivity of the RAAS (12). Psychosocial stress is likely capable of activating the RAAS by the following mechanisms: first, psychosocial stress activates the sympathetic adrenal medullary (SAM) system with consequent release of the catecholamines epinephrine and norepinephrine from the adrenal medulla (13). Catecholamines in turn induce renin synthesis and secretion via β-adrenergic receptors on juxtaglomerular cells in the kidney (4) resulting in further activation of the RAAS (1,4). The second mechanism comprises the hypothalamic pituitary adrenal (HPA) axis. Stress-induced secretion of hypothalamic corticotropin-releasing hormone (CRH) activates the pituitary to release adrenocorticotropic hormone (ACTH) into the circulation (13). Circulating ACTH stimulates the adrenal cortex to release not only cortisol in the zona fasciculata (13), but also aldosterone in the zona glomerulosa (12). Indeed, intravenous ACTH administration increased both cortisol and aldosterone concentrations in humans (12).

Studies investigating the reactivity of RAAS components to *mild acute mental stress,* notably without a psychosocial component and without a nonstress control group, provide mixed results with an overall tendency in support of RAAS activation. In reaction to cognitive tasks such as mental arithmetic or the Stroop test, plasma renin and/or plasma renin activity (PRA) were found to be either increased (14–20) or unchanged (21,22). Similarly, plasma angiotensin II increased (19) or remained unchanged (22), while aldosterone decreased (19) or remained unchanged (22). Examination as a non-standardized but potent *real-life* stressor induced substantial increases in PRA (23).

Notably, highest endocrine stress reactivity is usually observed in reaction to *psychosocial stress tasks* containing uncontrollable and social-evaluative elements (24) such as the Trier Social Stress Test (TSST) (25). Hitherto, human studies investigating reactivity of RAAS components to acute psychosocial stress have been scarce. In reaction to acute psychosocial stress by (modified) TSST, one pilot study without a nonstress control group measured in 9 healthy men plasma renin activity and plasma aldosterone before stress and up to 15 minutes after stress cessation (26). Results of that study point to a stress-induced increase in aldosterone but not renin after stress. In line with this, a recent comprehensive magnetic resonance imaging (MRI) study in healthy young men with intermediate TSST assessed 15 endocrine measures including serum aldosterone, but not renin, up to 115 minutes after stress and found aldosterone increases in reaction to TSST (27). Finally, one study investigated stress reactivity of salivary aldosterone in humans. In response to a modified TSST atopic patients and healthy controls showed increases in salivary aldosterone as compared to baseline assessment with highest levels observed 15 minutes after stress (28). In that study, aldosterone was measured up to 30 minutes after stress and salivary aldosterone levels did not complete recovery.

Taken together, the above-described evidence suggests that acute psychosocial stress is capable of activating the RAAS in humans. However, a systematic assessment of reactivity kinetics and interrelations of RAAS parameters (including simultaneous assessment of renin as first RAAS component and aldosterone as its end product) to standardized acute psychosocial stress induction as compared to a nonstress control group is lacking. Moreover, parallel assessment of plasma and salivary aldosterone stress responses is lacking and comparability thus remains unclear.

We therefore investigated whether acute psychosocial stress (induced by means of the highly potent TSST) compared with a nonstress control group activates the RAAS in healthy men. We aimed at identifying RAAS stress kinetics, including recovery, and interrelations of the measured parameters. We repeatedly measured renin and aldosterone, as well as HPA axis stress reactivity in terms of cortisol for validation and comparability purposes. Plasma measurements of renin and aldosterone were complemented by assessment of aldosterone in saliva.

## Methods

### Study participants

We intentionally recruited healthy, medication-free, nonsmoking, young men (18–30 years). Specific exclusion criteria as verified in a telephone interview by self-report included regular, occasional, or acute intake of medication, prior exposure to the TSST, excessive physical exercise, alcohol and illicit drug abuse, acute or chronic psychiatric or somatic disorders such as hypertension, any heart disease, varicosis, and thrombotic diseases, elevated blood sugar and diabetes, elevated cholesterol, coagulation disorder, liver and renal diseases, chronic obstructive pulmonary diseases, allergies and atopic diathesis, autoimmune diseases, gastrointestinal diseases, arthropathy or muscle disorders, human immunodeficiency virus, cancer, chronic pain, sleep disturbances, thyroid disease, and current infectious diseases. If the personal history was not conclusive, the participants’ primary care physician was contacted for clarification.

The Ethics Committee of the University of Constance, Germany, formally approved the research protocol. The study was carried out in accordance with the Declaration of Helsinki principles. Recruitment was carried out through online requests and advertisements at the University of Constance. All participants provided written informed consent before participating.

### Study design and procedure

We used a placebo-controlled, single-blind, between-subjects design. Eligible participants were assigned to either the experimental condition or the control condition. To age-match the 2 experimental groups a first person was randomly assigned to one group, and a second person of similar age (± 1 year) was assigned to the corresponding other group.

Participants were asked to abstain from vaccination for 4 weeks and from dental surgery for 2 weeks prior to the experimental session. For 48 hours they abstained from strenuous physical activity and alcohol, and the day before the experiment from any kind of sports, chocolate, and caffeine consumption. In addition, participants were asked to keep a normal sleeping pattern, to get up between 7 and 8 am on the day of the experiment and to avoid sour, caffeinated, or sweet drinks.

Participants reported to the laboratory of the Biological Work and Health Psychology group at the University of Constance at 11 am, where they provided informed consent. Afterwards they received water and the first standardized meal consisting of 2 bottles of Fresubin® (Fresenius Kabi Germany GmbH, 200 mL and 300 kcal per bottle) and 1 piece of crispbread. Then, body height and weight were measured. A venous catheter (Vasofix® Safety Braunule green/white G 18, Fa. B. Braun, Melsungen, Germany) was inserted into the nondominant arm about 30 minutes after arrival, followed about 50 minutes later by the first and thus baseline blood and saliva sampling (–1 minute). Then, the participants underwent the TSST or the placebo TSST (29) (see below). Immediately after the TSST or placebo TSST, a second blood and saliva sample was taken (+1 minute), followed by further samplings 10, 20, 30, 45, 60, 90, 120, and 180 minutes later, rendering a total of 10 sampling timepoints. Blood pressure (BP) was measured under resting conditions 10 minutes before catheter insertion and immediately before the first blood and saliva sampling by sphygmomanometry (Omron, M6, Nufringen, Germany). Resting mean arterial blood pressure (MAP) was calculated by the formula (2/3 × mean diastolic BP) + (1/3 × mean systolic BP).

Participants received a second meal at 3 pm, consisting of 2 bottles of Fresubin, 1 apple, and 1 piece of crispbread. At the end of the study day the participants were informed about the purpose of the study and were financially compensated (10€/hour).

### Psychosocial stress induction

To elicit a psychosocial stress response, we used the standard protocol of the widely used TSST, which combines a short introduction phase followed by a 3-minute preparation phase, a 5-minute mock job interview, and a 5-minute mental arithmetic task in front of an audience with video and auditory recording. The TSST evokes reliable increases in stress hormones (11,24). After the TSST, participants remained seated in a quiet room.

 The control group underwent a placebo version of the TSST (placebo TSST) that was structured corresponding to the TSST (3-minute preparation period, 5 minutes of free speech, and 5-minute mental arithmetic task) but without uncontrollability and social-evaluative threat (29).

## Endocrine Analyses

### Aldosterone

#### Plasma aldosterone.

Plasma aldosterone was measured in all participants at all blood sampling timepoints. Venous blood was drawn in ethylenediamine tetra-acetate (EDTA)-coated monovettes (Sarstedt AG & Co., Nümbrecht, Germany), and immediately centrifuged for 10 minutes at 2000g and 4°C. Obtained plasma was stored at –80°C until analysis. Plasma aldosterone levels were measured using a commercial enzyme-linked immunosorbent assay (ELISA, “Aldosterone ELISA,” RE-52301, IBL International GmbH, Hamburg, Germany) according to the manufacturer’s specifications. Optical density was measured using a microtiter plate reader (Synergy H1 Multi-Mode Microplate Reader, BioTek Instruments Inc., Bad Friedrichshall, Germany). Detection limit was <12.07 pg/mL. Mean inter- and intra-assay coefficients of variance (CVs) were 6.7% and 4.9%, respectively.

#### Salivary aldosterone.

Salivary aldosterone was measured in the last 23 participants of the stress group and the last 15 participants of the placebo TSST group at all saliva sampling timepoints. Saliva samples for measurement of aldosterone levels were collected using Salivette® devices (Sarstedt, Nümbrecht, Germany), which were stored at –20°C until analysis. Thawed saliva samples were centrifuged at 2500g for 10 minutes (Benchtop centrifuge: Megafuge 40R, Heraeus, Thermo Fischer Scientific, Langenselbold). Saliva aldosterone was measured using the same ELISA as plasma aldosterone (“Aldosterone ELISA,” RE-52301, IBL International GmbH, Hamburg, Germany) using 50 µL of saliva instead of plasma. Plasma and saliva samples of a participant were run simultaneously on the same ELISA plate. Mean inter- and intra-CVs of saliva aldosterone measurements were 6.7% and 5.5%, respectively.

#### Renin.

Renin was measured in all participants at 6 blood sampling timepoints (–1 minute, +1 minutes, +10 minutes, +20 minutes, +30 minutes, +60 minutes). We determined active renin from EDTA plasma by means of ELISA, according to the manufacturer’s instructions (“Renin (active) ELISA,” RE 53321, IBL International GmbH, Hamburg, Germany). Detection limit was 4.31 pg/mL. Mean inter- and intra-assay CVs were 5.7% and 5.2%, respectively.

#### Cortisol.

Cortisol was measured in all participants at 7 saliva sampling timepoints (-1 minute, +1 minute, +10 minutes, +20 minutes, +30 minutes, +45 minutes, +60 minutes). Cortisol was measured from the same salivettes as aldosterone. Salivary free cortisol was determined using an ELISA according to manufacturer’s protocol (“Cortisol Saliva ELISA,” RE-52611, IBL International GmbH, Hamburg, Germany). Detection limit was 0.003 μg/dL. Inter- and intra-assay CVs are below 9.3% and 7.3% according to the manufacturer’s information (IBL International GmbH, Hamburg, Germany).

For a more detailed description of aldosterone and renin assays, see the supplementary material provided in an online repository (30).

### Psychological assessment

Psychological assessment was performed to test for potential group differences. To assess the “big five” personality factors (neuroticism, extraversion, openness, agreeableness, and conscientiousness) we applied the 21-item version of the Big Five Inventory (BFI-K; 31) based on a 5-point rating scale (1 = “disagree strongly” to 5 = “agree strongly”) rendering scores from 1 to 5, with higher scores indicating higher levels of the respective personality factor. The data of 4 TSST participants and 2 placebo TSST participants were missing due to incompletion. Internal consistency of the 5 scales as depicted by Cronbach’s α coefficients ranged in our sample from .64 to .83.

To assess chronic stress we used the 12-item Chronic Stress Screening Scale from the Trier Inventory for Chronic Stress (TICS-CSSS; 32). The CSSS includes items about frequency of experiencing work overload (4 items), worries (4 items), lack of social recognition (2 items), excessive demands at work (1 item), and social overload (1 item) on a 5-point rating scale (0 = “never” to 4 = “very often”). Possible scores range from 0 to 48 with higher scores indicating a higher amount of chronic stress. The data of 1 TSST participant are missing due to incompletion. The global score of our sample showed a high internal consistency with Cronbach’s α = .90.

### Statistical analyses

Data were analyzed using SPSS (version 25.0) software packages (SPSS Inc., Chicago Il, USA). Data are presented as mean ± standard error of the mean (SEM). Tests were two-tailed with significance level at *P* ≤ .05. G*power 3.1 (33) analysis suggested that a total sample size of 58 is needed to detect an interaction between group and repeated endocrine parameters with an expected small effect size varying between *f* = .10 (renin, 6 repetitions, observed minimum intercorrelation among repeated measures of *r* = .74, and a power of .80) and *f* = .07 (aldosterone, 10 repetitions, observed minimum intercorrelation among repeated measures of *r* = .83, and a power of .82) in general linear models with repeated measures with α = .05. All data were tested for normal distribution and homogeneity of variance using Kolmogorov-Smirnov and Levene’s tests before statistical procedures were applied. Deviations from normality were considered when selecting statistical procedures (34).

Missing data were list-wise excluded for the respective parameter or replaced if possible as described below. We applied Huynh-Feldt correction for repeated measures to protect against violations of sphericity. Body mass index (BMI) was calculated with the formula BMI = kg/m^2^.

We compared groups in terms of demographic, physiological, and psychological measures, as well as baseline levels of endocrine measures using univariate analyses of variance (ANOVA). To test whether the TSST compared with the placebo-TSST induced significant changes in endocrine parameters we calculated repeated measures analyses of (co)variance (AN(C)OVA) with group as the independent variable and repeated plasma aldosterone, saliva aldosterone, plasma renin, and salivary cortisol levels as dependent variables. Owing to effects on stress reactivity we performed all AN(C)OVA analyses without and with controlling for age, BMI, and MAP as potentially confounding covariates (10). Post hoc testing of significant stress effects in endocrine measures comprised univariate ANOVAs with group as the independent variable and single endocrine measurements as dependent variables. Moreover, to identify significant changes from baseline, we calculated for each endocrine parameter separate in stress and control group repeated ANOVAs with baseline and separate post-stress measurements as repeated dependent variable.

To test for associations between stress reactivity of the different endocrine measures, we calculated Spearman’s rank-order correlations. Correlation analyses were performed between areas under the curve with respect to increase (AUCi) (35) for plasma and saliva aldosterone, renin, and cortisol as well as between the respective stress increase scores (ie, from baseline to peak).

Effect size parameters (*f*) were calculated from partial eta squared (η _*p*_^2^) and are reported where appropriate (effect size conventions: η _*p*_^2^: > .01 = small, > .06 = moderate, > .14 = large; *f*: .10 = small, .25 = medium, .40 = large) (36).

Out of 580 samples in total (58 participants, each with 10 samples) 15 missing post-stress samples for endocrine measurements within the recovery period after the peak, were replaced by the mean of the respective preceding and subsequent measurements (if available), or the respective preceding or subsequent measurement to prevent listwise exclusion of the respective participant and maximize statistical power.

## Results

### Subjects’ characteristics

To account for expected dropouts due to problems with blood sampling we recruited a total of 70 participants with baseline blood sampling after successful venous catheter insertion, with 40 participants in the TSST group and 30 in the placebo TSST group. Owing to venous catheter occlusion and/or severe problems with blood sampling 12 participants had to be excluded. The final study sample comprised 58 participants with 34 participants in the TSST group and 24 in the placebo TSST group. We obtained plasma aldosterone and salivary cortisol levels of all 58 participants. Plasma renin measurements were missing in one participant of the TSST group due to technical problems (*N* = 57). The final saliva aldosterone sample comprised 23 participants in the TSST group and 15 in the placebo-TSST group (*N* = 38). The reason for this reduced sample size is that the salivary aldosterone assay was implemented at a later stage of the study when salivary cortisol analyses of the first 20 study subjects had already been performed and their saliva samples had been thawed once and then stored again at –20°C. We analyzed salivary aldosterone from repeatedly thawed saliva samples of 6 of these participants and found that their salivary aldosterone levels were substantially lower than their plasma aldosterone levels (data not shown). To not compromise the reliability of salivary aldosterone assessment, we decided to restrict salivary aldosterone analyses to samples that had not been thawed before. Missing questionnaire data were listwise excluded.

Participants’ characteristics are depicted in Table 1. The groups did not significantly differ in age, BMI, MAP, endocrine measures at baseline, or psychological variables (all *P* > .11)

**Table 1.** Characteristics of Study Subjects

	TSST (*N* = 34) *mean* ± *SEM* (*R*)	Placebo-TSST (*N* = 24) *mean* ± *SEM* (*R*)	*P*
Age (years)	23.24 ± 0.40 (18–27)	23.54 ± 0.51 (20–29)	.64
BMI (kg/m^2^)	24.23 ± 0.53 (18.37–33.19)	23.44 ± 0.46 (20.48–28.55)	.29
MAP (mmHg)	90.91 ± 1.13 (79.56–106.78)	94.55 ± 2.10 (71.67–111.67)	.11
Plasma aldosterone (pg/mL)	110.80 ± 12.79 (29.28–284.27)	103.47 ± 12.81 (39.87–285.44)	.70
Saliva aldosterone (pg/mL)	93.59 ± 8.63 (49.59–204.45), *n* = 23	95.11 ± 11.72 (42.07–235.77), *n* = 15	.92
Plasma renin (pg/mL)	32.43 ± 2.73 (8.16–81.04), *n* = 33	25.70 ± 3.38 (5.98–81.96)	.12
Cortisol (nmol/)	5.72 ± 0.59 (1.68–20.53)	7.89 ± 1.33 (1.57–29.67)	.11
BFI-K	*n* = 30	*n* = 22	
Extraversion	3.62 ± 0.15 (1.75–4.75)	3.24 ± 0.20 (2.00–5.00)	.13
Agreeableness	2.93 ± 0.13 (1.25–4.50)	2.94 ± 0.17 (1.50–4.50)	.96
Neuroticism	2.63 ± 0.16 (1.00–4.50)	2.38 ± 0.18 (1.25–4.25)	.30
Openness	3.58 ± 0.15 (2.20–5.00)	3.43 ± 0.15 (2.20–4.80)	.49
Conscientiousness	3.40 ± 0.11 (1.75–4.50)	3.49 ± 0.15 (2.25–4.75)	.62
TICS-CSSS	17.94 ± 1.63 (3–42), *n* = 33	17.38 ± 1.84 (6–40)	.82

Abbreviations: *N*, total number of participants; *n*, number of participants in case of missing data; SEM, standard error of the mean; R, range; BMI, body mass index; MAP, mean arterial pressure; BFI-K, Big Five Inventory Short Version; CSSS, Chronic Stress Screening Scale from the Trier Inventory for Chronic Stress.

### Stress reactivity

#### Plasma aldosterone.

**Figure 1.**
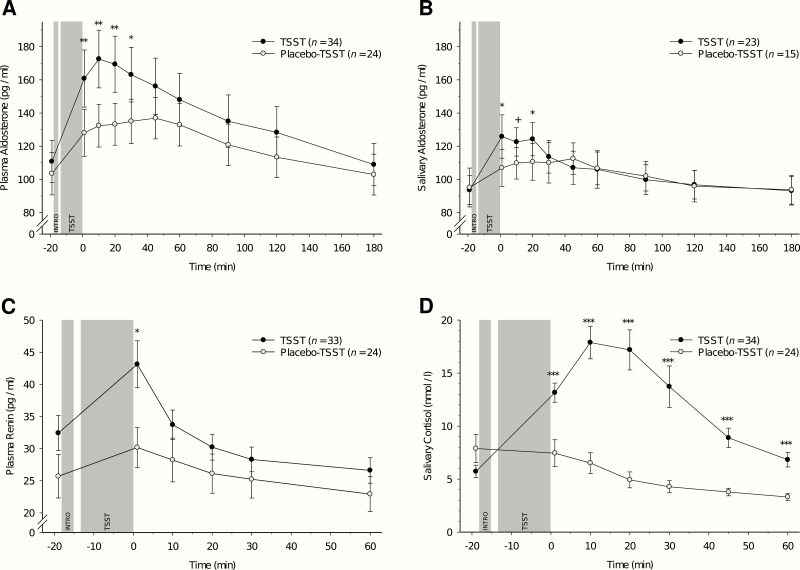
Responses in endocrine measure levels to Trier Social Stress Test (TSST) and placebo-TSST (mean ± SEM). (A) Plasma aldosterone. (B) Salivary aldosterone. (C) Plasma renin. (D) Salivary cortisol. Asterisks indicate significant group differences between TSST and placebo-TSST independent of the respective baseline levels (*P < .05; **P < .01; ***P < .001; +*P* < .10).

General linear models with repeated measures revealed that as compared to the nonstress control group, stress induction by TSST induced significant increases in plasma aldosterone (interaction group-by-time: *F*(3.14, 175.93) = 4.32, *P* = .005, η _*p*_^2^ = .07, *f* = .28) with highest levels 10 and 20 minutes after TSST cessation (Fig. 1A). Controlling for potential confounding variables (age, BMI, and MAP) did not significantly change these results (*P* = .021).

Post hoc testing indicated significant group differences during the first 30 minutes after stress/placebo-stress cessation while controlling for the baseline measurement (+1 minute, *F*(1, 55) = 10.81, *P* = .002, η _*p*_^2^ = .16, *f* = .44; +10 minutes, *F*(1, 55) = 11.46, *P* = .001, η _*p*_^2^ = .17, *f* = .46; +20 minutes, *F*(1, 55) = 9.81, *P* = .003, η _*p*_^2^ = .15, *f* = .42; and +30 minutes, *F*(1, 55) = 5.88, *P* = .019, η _*p*_^2^ = .10, *f* = .33). The groups did not differ at later timepoints (45 minutes to 180 minutes after TSST cessation: all *P* ≥ .24). Further post hoc testing comprised separate reanalyses in the TSST group only. Compared with baseline, significantly increased plasma aldosterone levels were observed at all post-stress measurement timepoints up to 2 hours after stress cessation (1 minute to 120 minutes: all *P *≤ .011, see Table 2). The last measurement (+180) did not significantly differ from the baseline (*P* = .72). Separate reanalyses in the placebo TSST group only revealed that compared with baseline, plasma aldosterone levels were significantly increased at all post-treatment measurement timepoints up to 1.5 hours after stress cessation (1 minute to 90 minutes: all *P* ≤ .009, see Table 2). The last two measurements (+120 and +180) did not significantly differ from the baseline (all *P* ≥ .11).

**Table 2.** Post hoc test results for the TSST and placebo-TSST group separately: comparisons between baseline and each post-stress measurement

	TSST				Placebo-TSST			
	*(df*_*Num*_, *df*_*Den*_)	*F*	*P*	η_*p*_^2^*/f*	*(df*_*Num*_ *, df*_*Den*_)	*F*	*P*	η_*p*_^2^*/f*
**Plasma aldosterone**								
+1 minute	(1, 33)	70.29	<.001	.68 / 1.46	(1, 23)	25.87	<.001	.53 / 1.06
+10 minutes	(1, 33)	68.51	<.001	.68 / 1.44	(1, 23)	39.58	<.001	.63 / 1.30
+20 minutes	(1, 33)	80.68	<.001	.71 / 1.56	(1, 23)	27.12	<.001	.54 / 1.08
+30 minutes	(1, 33)	78.71	<.001	.71 / 1.55	(1, 23)	31.83	<.001	.58 / 1.18
+45 minutes	(1, 33)	50.42	<.001	.60 / 1.24	(1, 23)	25.60	<.001	.53 / 1.06
+60 minutes	(1, 33)	43.33	<.001	.57 / 1.15	(1, 23)	18.08	<.001	.44 / .89
+90 minutes	(1, 33)	13.69	<.001	.29 / .64	(1, 23)	8.22	.009	.26 / .59
+120 minutes	(1, 33)	7.20	.011	.18 / .47	(1, 23)	2.84	.11	
+180 minutes	(1, 33)	0.13	.72		(1, 23)	0.01	.92	
**Salivary aldosterone**								
+1 minute	(1, 22)	26.30	<.001	.55 / 1.09	(1, 14)	9.85	.007	.41 / .84
+10 minutes	(1, 22)	34.03	<.001	.61 / 1.24	(1, 14)	7.32	.017	.34 / .72
+20 minutes	(1, 22)	39.55	<.001	.64 / 1.34	(1, 14)	14.33	.002	.51 / 1.01
+30 minutes	(1, 22)	25.63	<.001	.54 / 1.08	(1, 14)	10.62	.006	.43 / .87
+45 minutes	(1, 22)	19.68	<.001	.47 / .95	(1, 14)	6.70	.021	.32 / .69
+60 minutes	(1, 22)	5.29	.031	.19 / .49	(1, 14)	2.88	.11	
+90 minutes	(1, 22)	3.04	.10		(1, 14)	1.32	.27	
+120 minutes	(1, 22)	0.62	.44		(1, 14)	0.01	.92	
+180 minutes	(1, 22)	0.01	.93		(1, 14)	0.04	.85	
**Plasma renin**								
+1 minute	(1, 32)	17.73	<.001	.36 / .73	(1, 23)	11.88	.002	.34 / .72
+10 minutes	(1, 32)	0.53	.47		(1, 23)	8.48	.008	.27 / .61
+20 minutes	(1, 32)	2.19	.15		(1, 23)	0.23	.64	
+30 minutes	(1, 32)	6.80	.014	.18 / .46	(1, 23)	0.20	.66	
+60 minutes	(1, 32)	15.28	<.001	.32 / .69	(1, 23)	4.86	.038	.18 / .20
**Salivary cortisol**								
+1 minute	(1, 33)	54.70	<.001	.62 / 1.29	(1, 23)	0.46	.51	
+10 minutes	(1, 33)	51.11	<.001	.61 / 1.25	(1, 23)	5.46	.029	.19 / .49
+20 minutes	(1, 33)	30.45	<.001	.48 / .96	(1, 23)	16.67	<.001	.42 / .85
+30 minutes	(1, 33)	14.52	<.001	.31 / .66	(1, 23)	18.78	<.001	.45 / .90
+45 minutes	(1, 33)	8.12	.007	.20 / .50	(1, 23)	15.24	.001	.40 / .81
+60 minutes	(1, 33)	1.53	.23		(1, 23)	13.93	.001	.38 / .78

*df*_*Num*_ = degrees of freedom numerator, *df*_*Den*_* = *degrees of freedom denominator.

Statistically significant results are highlighted in bold.

#### Salivary aldosterone.

 Stress induction by TSST induced significant increases in salivary aldosterone (group-by-time-interaction: *F*(4.90, 176.41) = 2.38, *P* = .041, η _*p*_^2^ = .06, *f* = .26), with highest levels at 1, 10, and 20 minutes after TSST cessation (Fig. 1B). Controlling for potential confounding variables (age, BMI, and MAP) slightly reduced the significance level of this interaction effect to marginal significance (*P* = .066).

Post hoc testing revealed group differences during the first 20 minutes after TSST/placebo TSST cessation while controlling for the baseline measurement (+1 minute, *F*(1, 35) = 6.50, *P* = .015, η _*p*_^2^ = .16, *f* = .43; +10 minutes, *F*(1, 35) = 4.05, *P* = .052, η _*p*_^2^ = .10, *f* = .34; and +20 minutes, *F*(1, 35) = 4.72, *P* = .037, η _*p*_^2^ = .12, *f* = .37). The groups did not significantly differ during the remaining later measurements (30 minutes to 180 minutes after TSST cessation: all *P *≥ .41). Again, further post hoc testing comprised separate reanalyses in the TSST group. Compared with baseline, significantly increased salivary aldosterone levels were observed during the first hour after TSST (1 minute to 60 minutes: all *P* ≤ .031, see Table 2). The subsequent measurements (90 minutes to 180 minutes) did not significantly differ from baseline (all *P* ≥ .10). Separate reanalyses in the placebo-TSST group only revealed that, compared with baseline, salivary aldosterone levels were significantly increased at post-stress measurement timepoints up to 1 hour after stress cessation (1 minute to 45 minutes: all *P* ≤ .021, see Table 2). Subsequent measurements (60 minutes to 180 minutes) did not significantly differ from baseline (all *P* ≥ .11).

#### Plasma renin.

Stress induction by TSST induced significant increases in plasma renin as compared to the control group (group-by-time-interaction: *F*(2.47, 135.66) = 5.15, *P* = .004, η _*p*_^2^ = .09, *f* = .31) with highest levels at +1 minute after TSST cessation (Fig. 1C). This interaction was independent of the potential confounders age, BMI, and MAP (*P* = .017).

Post hoc testing showed significant group differences at +1 minute after stress/placebo (*F*(1, 54) = 4.31, *P* = .043, η _*p*_^2^ = .07, *f* = .28) independent of baseline assessment. The groups did not differ in their later measurements (+10 minutes, +20 minutes, +30 minutes, and +60 minutes after TSST/placebo TSST cessation, all *P* ≥ .34). As depicted in Table 2, further post hoc testing in the TSST group only revealed that as compared to baseline, significantly increased plasma renin concentrations were observed at +1 minute (*P* < .001), while the measurements +10 and +20 minutes after TSST did not significantly differ from baseline (all *P* ≥ .15). Decreased renin levels were observed +30 minutes (*P* = .014), and +60 minutes (*P *< .001) after stress cessation. Separate reanalyses in the placebo-TSST group only revealed that as compared to baseline, plasma renin levels were significantly increased at the first 2 post-stress measurement timepoints (+1 minute, *P* = .002; +10 minutes, *P* = .008). The subsequent measurements +20 and +30 minutes after TSST cessation did not significantly differ from baseline (all *P* ≥ .64) while the last measurement (+60) was significantly reduced (*P* = .038).

#### Salivary cortisol.

Compared with the placebo TSST group, the TSST group showed significant increases in salivary cortisol (group-by-time-interaction: *F*(2.93, 164.06) = 16.82, *P* < .001, η _*p*_^2^ = .23, *f* = .55), with highest salivary cortisol levels in the TSST group at +10 minutes and +20 minutes after TSST (Fig. 1D). Controlling for confounders (age, BMI, and MAP) did not change results (*P* < .001). Higher cortisol levels in the TSST group than in the control group were observed during 60 minutes after treatment cessation independent of baseline assessment (+1 minute, +10 minutes, +20 minutes, +30 minutes, +45 minutes, +60 minutes, all *P* < .001, all η _*p*_^2^ ≥ .23, all *f* ≥ .54). Compared with baseline, salivary cortisol levels were significantly increased during the first 45 minutes after TSST (1 minute to 45 minutes: all *P* ≤ .007, see Table 2). The measurement +60 minutes after TSST cessation did not significantly differ from the baseline (*P* = .23). In the placebo TSST group there was no significant difference from baseline immediately after stress cessation (*P* = .51), while all subsequent measurements (10 minutes to 60 minutes) were significantly reduced (all *P* ≤ .029).

### Associations between aldosterone, renin, and cortisol

Correlations between AUCi of the different endocrine measures are depicted in Table 3, while Table 4 depicts correlations for stress increase scores.

**Table 3.** Correlations (r) between AUCi for plasma aldosterone, salivary aldosterone, plasma renin, and salivary cortisol in the TSST group

	Salivary aldosterone	Plasma renin	Salivary cortisol
Plasma aldosterone	**.70, *P* < .001, *n = *23**	.28, *P = *.11, *n = *33	.14, *P = *.42, *n = *34
Salivary aldosterone		.18, *P = *.41, *n = *23	.14, *P = *.54, *n = *23
Plasma renin			**.35, *P = *.047, *n = *33**

Abbreviation: *n*, number of participants for the respective calculation, statistically significant results are highlighted in bold.

**Table 4.** Correlations (r) between stress increase scores for plasma aldosterone, salivary aldosterone, plasma renin, and salivary cortisol in the TSST group

	Salivary aldosterone	Plasma renin	Salivary cortisol
Plasma aldosterone	**.63, *P = *.001, *n = *23**	**.36, *P = *.039, *n = *33**	**.52, *P = *.002, *n = *34**
Salivary aldosterone		**.65, *P < *.001, *n = *23**	**.58, *P = *.004, *n = *23**
Plasma renin			**.36, *P = *.038, *n = *33**

Abbreviation: *n*, number of participants for the respective calculation, statistically significant results are highlighted in bold.

#### AUCi.

Higher plasma aldosterone AUCi significantly related to higher salivary aldosterone AUCi (*r* = .70, *P* < .001). Moreover, higher plasma renin AUCi was associated with higher cortisol AUCi (*r* = .35, *P* = .047). Neither plasma nor salivary aldosterone AUCi were significantly related to renin or cortisol AUCi (all *P* ≥ .11).

#### Stress increase scores.

With respect to stress increase scores we found all endocrine parameters to correlate with each other: higher plasma aldosterone stress increase scores correlated significantly with higher salivary aldosterone (*r* = .63, *P* = .001), higher plasma renin (*r* = .36, *P* = .039), and higher cortisol stress increase scores (*r* = .52, *P* = .002). Moreover, higher salivary aldosterone stress increase scores were associated with higher plasma renin (*r* = .65, *P* < .001) and higher salivary cortisol stress increase scores (*r* = .58, *P* = .004). Finally, plasma renin stress changes were significantly related to cortisol stress changes (*r* = .36, *P* = .038).

## Discussion

The main objective of the study was the systematic investigation and characterization of stress reactivity kinetics and stress recovery of RAAS parameters measured in plasma and saliva as well as their interrelations and associations with cortisol. We measured plasma and salivary aldosterone, plasma renin, and salivary cortisol up to 3 hours after acute psychosocial stress compared with placebo stress to fully cover stress reactivity and recovery. The main finding of our study is that acute psychosocial stress increases renin and aldosterone compared with a nonstress control condition with medium to large effects. More precisely, we observed in reaction to stress immediate increases in plasma renin that quickly returned to baseline levels within 10 minutes, and that even decreased starting from 30 minutes after stress. For aldosterone we observed later and longer lasting increases, with highest levels during the 20 minutes after stress, both for plasma and salivary aldosterone. For plasma aldosterone, it took 3 hours until baseline levels were reached again, while salivary aldosterone recovered more quickly within 90 minutes after stress. We moreover observed the well-documented stress reactivity pattern of cortisol with peak reactivity 10 and 20 minutes after TSST cessation and recovery completed 1 hour after stress (eg, (11)).

With respect to *temporal sequence* our findings suggest that acute psychosocial stress activates the RAAS, starting with renin increases followed by salivary and plasma aldosterone increases in addition to cortisol secretion. Although to the best of our knowledge, stress-induced increases of both renin and aldosterone, as well as parallel assessment of plasma and salivary aldosterone in humans have not yet been reported, our findings are in line with the literature. For instance, cognitive stress tasks (14–20) and examination stress (23) induced increases in renin and/or PRA. Moreover, pilot data (*n* = 9) and data from one patient and one MRI study suggest that as compared to baseline levels, psychosocial stress induces increases in aldosterone measured either in plasma (26), serum (27), or saliva (28). Potential preceding increases in PRA were investigated in the pilot study only, but results remained unclear (26).

With respect to *stress kinetics*, most studies investigating PRA/plasma renin in reaction to stress observed in line with our findings immediate stress increases (14–18,20). Renin recovery had so far been studied in a pilot study in 6 participants where recovery was terminated within 15 minutes after peak reactivity observed 15 minutes after a mental arithmetic task (19). Our finding of highest aldosterone stress increases 10 and 20 minutes after TSST cessation is in line with previous, but notably only descriptive observations, as required statistics have not been reported (26–28). While two of these studies did not assess a sufficient time interval to allow recovery (26,28), one study assessed serum aldosterone up to 115 minutes after stress cessation but the recovery period had been compromised by a 30-minute MRI assessment in supine position instead of regular resting in sitting position (27). Results of that study may thus not be generalized to recovery under nonsupine posture conditions and recovery had not been explicitly addressed. While in our study plasma aldosterone and cortisol showed similar peak reactivity patterns, salivary aldosterone started earlier, at 1 minute after stress, but remained elevated for a comparable period. However, given the lower sample size in salivary aldosterone, and given the high correlation of *r* = .70 between stress reactivity (AUCi) of plasma and salivary aldosterone, we feel that a potentially faster increase in salivary aldosterone should be interpreted with caveat until further confirmation by future research. The observed high correlations between plasma and salivary aldosterone are in line with previous research under nonstress conditions (37).

Given this and based on our interrelation analysis with stress-induced renin increases relating to increases in both plasma and salivary aldosterone, we propose with respect to *underlying mechanisms* that renin stress increases likely stimulate subsequent aldosterone release. This corresponds with the literature on renin as a well-recognized stimulant of angiotensin and ultimately, aldosterone (1,2). Additionally, studies examining temporal profiles of renin and aldosterone showed significant associations between both hormones with time lag up to 20 minutes between renin/PRA increases and subsequent aldosterone increases (eg, 38,39). Whether the earlier renin stress increase also relates to the subsequent cortisol stress response is less clear despite the observed significant positive correlation. A potential underlying mechanism is that cortisol is indirectly stimulated by renin via renin-induced stimulation of ANG-II (1) that in turn induces ACTH release (40) and consequently cortisol secretion (13). An ANG-II mediated direct cortisol stimulation is unlikely (41,42). Alternatively, a parallel activation of cortisol (13) and renin (43,44) via stress-induced ACTH secretion seems possible despite contradicting results (45,46). The immediate stress reactivity peak in renin likely results from stress-induced immediate SAM axis and sympathetic nervous system (SNS) activation in reaction to stress induction. Indeed, catecholamine peak reactivity following TSST is usually observed immediately after stress (11,13) and thus at the same time like renin in the present study. In addition to stress-induced renin increases, aldosterone stress reactivity supposedly results from concomitant direct stimulation by stress-induced ACTH release from the anterior pituitary as ACTH administration increased plasma aldosterone and cortisol concentrations (12). As both cortisol and aldosterone are produced in the adrenal cortex it seems plausible that it takes a similar time for pituitary ACTH to reach all zones of the adrenal cortex. Indeed, cortisol secretion begins about 10 minutes after ACTH rise in plasma (47) and aldosterone responses have comparably been observed about 15 minutes after ACTH increase (48).

Taken together, the proposed mechanistic sequence of events from stress exposure to aldosterone release is as follows: stress-induced immediate SAM axis and SNS activation in addition to ACTH release stimulate renin. Renin in turn induces, via angiotensinogen and ANG-I, ANG-II release resulting in aldosterone secretion. Aldosterone secretion is further costimulated by stress-induced immediate ACTH release. Notably, as we did neither measure catecholamines or ACTH, this proposed sequence of events in reaction to stress warrants confirmation by future research.

We can only speculate whether our findings have clinical implications with respect to stress-related cardiovascular risk. Elevated basal renin and/or aldosterone levels have been observed in hypertensive patients and other CVD related outcomes (see opening paragraph) and regular medical treatment for CVD patients comprises drugs that inhibit or down-regulate RAAS activation (eg, renin inhibitors, angiotensin receptor blockers, ACE inhibitors, MR antagonists for aldosterone inhibition) (2–4). Given that acute stress has been shown to trigger acute coronary syndromes (ACS) in vulnerable patients within 2 hours (8), a stress-induced transient RAAS activation that in terms of aldosterone lasts for 2 hours or more may represent a mechanism that adds to ACS triggering. Moreover, given the role of the RAAS in blood pressure regulation (1–4), stress-induced transient RAAS activation may relate to hypertension and elevated CVD risk. In line with the allostatic load concept by McEwen (49) it is conceivable that under conditions of chronic stress the observed acute stress increases in renin and/or aldosterone may accumulate over time to chronically elevated levels and consequently elevated (CVD) disease risk. While associations between chronic psychosocial stress and RAAS components have hardly been studied in humans, studies in rodents are in support of elevated renin (50–54) and/or aldosterone levels with chronic stress (53), although results are in part inconclusive (51,52,54). In humans, increased basal renin and/or plasma aldosterone concentrations have been observed in post-traumatic stress disorder (55,56) and depression (57) as psychiatric disorders discussed in the context of chronic stress. It is also conceivable that chronic stress either ultimately or depending on its nature leads to blunted RAAS activity as has been discussed for HPA axis stress reactivity (58), in particular in the context of vital exhaustion (59,60), and with respect to the allostatic load type “inadequate response” (49).

Our finding that assessment of plasma aldosterone (that notably requires invasive blood sampling with its well-known consequences) could be substituted by non-invasive reliable and valid salivary aldosterone assessment may have implications for future research.

Strengths of our study include the use of the TSST as a highly potent standardized laboratory stress test and our control condition “Placebo-TSST” that controls for body position and type of tasks. This is of particular importance given that body posture changes relate to increases in plasma aldosterone and plasma renin activity (61). Indeed, the placebo-TSST group also showed significant, albeit lower increases in renin and aldosterone compared with the TSST group. Another strength is that with a 3-hour post-stress assessment, we decided on a sufficiently long time interval to assess reactivity kinetics completely, including stress increases and recovery. Further strengths comprise the parallel assessment of plasma and salivary aldosterone, random group assignment and subsequent age matching, and the recruitment of healthy, non-smoking, drug-free men. Finally, we standardized meal and water intake as aldosterone and renin are sensitive to fluid and food intake (62,63). Our study also has limitations. The generalizability of the results is limited, as our study sample comprised healthy young men only. Whether our findings are generalizable to women, older and younger individuals, or patients with CVD remains unclear. Also, our sample size for salivary aldosterone was smaller than that of plasma aldosterone, which could have compromised salivary aldosterone stress reactivity kinetics including recovery. Moreover, our proposed causality assumptions need to be verified in experimental studies using stimulating and blocking agents.

In sum, we found acute stress to activate the RAAS as indicated by higher renin and aldosterone increases as compared to a nonstress control condition with earlier renin and later aldosterone reactivity. The high correlation between plasma and salivary aldosterone stress reactivity implicates that salivary aldosterone assessment could be taken into consideration as a valid noninvasive alternative. Future research is needed to address whether chronic psychosocial stress relates to potential RAAS adaptations and whether stress reactivity of RAAS components is associated with CVD risk. Moreover, determinants and modulating factors of the RAAS stress reactivity need to be elucidated.

## Abbreviations

ACE: angiotensin-converting enzyme
ACTH: adrenocorticotropic hormone
ANG-I: angiotensin I
BMI: body mass index
BP: blood pressure
CRH: corticotropin-releasing hormone
CVD: cardiovascular disease
HPA: hypothalamic pituitary adrenal
MAP: mean arterial blood pressure
MRI: magnetic resonance imaging
PRA: plasma renin activity
SAM: sympathetic adrenal medullary
TSST: Trier Social Stress Test

## Additional Information

***Disclosure Summary:*** The authors have nothing to disclose. There are no conflicts of interest.

***Data availability:*** The datasets generated during and/or analyzed during the current study are not publicly available but are available from the corresponding author on reasonable request.
